# Toolkit for cellular studies of mammalian mitochondrial inorganic polyphosphate

**DOI:** 10.3389/fcell.2023.1302585

**Published:** 2023-12-15

**Authors:** Vedangi Hambardikar, Yaw A. Akosah, Ernest R. Scoma, Mariona Guitart-Mampel, Pedro Urquiza, Renata T. Da Costa, Matheus M. Perez, Lindsey M. Riggs, Rajesh Patel, Maria E. Solesio

**Affiliations:** ^1^ Department of Biology, and Center for Computational and Integrative Biology (CCIB), Rutgers University, Camden, NJ, United States; ^2^ Department of Molecular Pathobiology, College of Dentistry, New York University, New York City, NY, United States; ^3^ Robert Wood Johnson Medical School, Rutgers University, New Brunswick, NJ, United States

**Keywords:** mitochondria, inorganic polyphosphate, polyP, mammalian cells, bioenergetics, toolkit

## Abstract

**Introduction:** Inorganic polyphosphate (polyP) is an ancient polymer which is extremely well-conserved throughout evolution, and found in every studied organism. PolyP is composed of orthophosphates linked together by high-energy bonds, similar to those found in ATP. The metabolism and the functions of polyP in prokaryotes and simple eukaryotes are well understood. However, little is known about its physiological roles in mammalian cells, mostly due to its unknown metabolism and lack of systematic methods and effective models for the study of polyP in these organisms.

**Methods:** Here, we present a comprehensive set of genetically modified cellular models to study mammalian polyP. Specifically, we focus our studies on mitochondrial polyP, as previous studies have shown the potent regulatory role of mammalian polyP in the organelle, including bioenergetics, via mechanisms that are not yet fully understood.

**Results:** Using SH-SY5Y cells, our results show that the enzymatic depletion of mitochondrial polyP affects the expression of genes involved in the maintenance of mitochondrial physiology, as well as the structure of the organelle. Furthermore, this depletion has deleterious effects on mitochondrial respiration, an effect that is dependent on the length of polyP. Our results also show that the depletion of mammalian polyP in other subcellular locations induces significant changes in gene expression and bioenergetics; as well as that SH-SY5Y cells are not viable when the amount and/or the length of polyP are increased in mitochondria.

**Discussion:** Our findings expand on the crucial role of polyP in mammalian mitochondrial physiology and place our cell lines as a valid model to increase our knowledge of both mammalian polyP and mitochondrial physiology.

## Introduction

Inorganic polyphosphate (polyP) is a biopolymer comprised of multiple phosphate units (Pi) bound together by high-energy phosphoanhydride bonds, similar to those found in ATP (Morrissey et al., 2012). PolyP is present in every tissue from every studied organism, ranging from unicellular bacteria to higher eukaryotes (Kumble and Kornberg, 1995; Osorio et al., 2022). The physiological roles of polyP in prokaryotes and lower eukaryotes have been extensively studied. For example, it is known that polyP is crucial for bacterial growth and survival (Rao et al., 2009), as well as in the post-translational modification of proteins in yeast (via polyphosphorylation) (Azevedo et al., 2015). In mammals, polyP is involved in the regulation of multiple physiological processes at the cellular level. For example, its involvement in blood coagulation and inflammatory response (Smith et al., 2006; Mailer et al., 2019), bone and cartilage formation (Wang et al., 2019; Khong et al., 2020), energy metabolism (Gray and Jakob, 2015; Solesio et al., 2016a; Solesio et al., 2016b; Solesio et al., 2020; Solesio et al., 2021; Hambardikar et al., 2022), amyloidogenesis (Yoo et al., 2018; Lempart and Jakob, 2019; Xie and Jakob, 2019), and ion channel regulation (Laver et al., 2001; Zakharian et al., 2009; Stotz et al., 2014), has been demonstrated. Moreover, in the same organisms, polyP plays a role in carcinogenesis and tumorigenesis (Kulakovskaya et al., 2018; Boyineni et al., 2020), as well as in neurodegeneration (Borden et al., 2021; McIntyre and Solesio, 2021). However, the exact mechanism by which polyP exerts all these roles is still far from being fully understood.

The distribution of polyP is not uniform in mammalian cells, as it varies in different cell types. For example, using rats and mice, some authors showed that polyP is present in the brain, heart, kidneys, lungs, and liver of these animals; with subcellular presence in the nucleus, plasma membrane, cytosol, mitochondria, and microsomes (Kornberg et al., 1999). Moreover, we and others have described that, in mammalian cells, there is an especially high abundance of polyP in mitochondria (Abramov et al., 2007; Solesio et al., 2016b). In fact, the mammalian F_0_F_1_ ATP synthase is involved in the metabolism of polyP (even if other enzymes could also be implicated in this process) (Baev et al., 2020); and the levels of polyP are closely linked to the status of the mitochondrial respiratory chain (Seidlmayer et al., 2012a).

PolyP’s highly energetic bonds and mitochondrial abundance in mammalian cells suggest that this polymer could be involved in the maintenance of mitochondrial physiology in these cells. This possibility is supported by multiple results. For example, in a study conducted using cardiac myocytes, the authors demonstrated that polyP is a crucial regulator of the mitochondrial permeability transition pore (mPTP) (Seidlmayer et al., 2012a). These findings were corroborated in different cell lines, including HepG2, HEK293, and C2C12, along with primary cultures from astrocytes and neurons from rats (Abramov et al., 2007). Additionally, the role of polyP in the maintenance of proper mitochondrial calcium homeostasis has also been demonstrated (Solesio et al., 2016b; Solesio et al., 2020); and a proteomics and metabolomics study using SH-SY5Y cells revealed the involvement of polyP in many bioenergetics-related mammalian pathways (Guitart-Mampel et al., 2022). Finally, using HEK293 cells, we have demonstrated a shift in energy metabolism from mitochondrial to extra mitochondrial metabolic pathways (Solesio et al., 2021). Accordingly, in these cells, we found lower ATP levels; decreased mitochondrial oxidative phosphorylation (OXPHOS); and upregulation of cytosolic metabolic pathways; such as glycolysis (Solesio et al., 2021), and pentose phosphate pathway (Hambardikar et al., 2022). Accumulation of reactive oxygen species (ROS) and dysregulated antioxidant defense in response to decreased mitochondrial polyP levels were also reported (Hambardikar et al., 2022).

While the role of polyP in the regulation of mitochondrial bioenergetics seems clear, the mechanism(s) that underly this effect still remain unclear. One of the main reasons for this dearth of knowledge is that the metabolism of polyP is poorly understood in mammalian cells. However, this is not the case in prokaryotes and lower eukaryotes. For example, Kornberg et al., isolated, purified, and cloned two of the main enzymes involved in the metabolism of polyP in these organisms; which are, the polyphosphate kinase (PPK) and the exopolyphosphatase (PPX) (Kornberg et al., 1999). While PPK synthesizes polyP by the transfer of the terminal Pi of ATP to a polyP chain; PPX hydrolyzes polyP to Pi, at its terminal ends. Another enzyme involved in the metabolism of polyP, which was also initially described by Kornberg and his team, is the endopolyphosphatase (PPN). PPN internally cleaves long chain polyP into short and medium chains (Kornberg et al., 1999). While PPX and PPN are both polyP hydrolyzing enzymes, the length of the chain of polyP produced by these enzymes could be significantly different. It is important to note that PPX can act in both long and short chain polyP, while PPN shows a higher substrate specificity towards longer chains (more than 60 Pi) of polyP (Andreeva et al., 2019). All these enzymes have been identified in various microorganisms and lower eukaryotes. However, no homologs have been found so far in mammals, despite multiple efforts conducted by different research groups. Additional complexity to polyP studies in mammalian cells is added by the lack of well-described and comprehensive methods to extract and quantify polyP, even though recently some significant advancement have been published in this field, mostly in other organisms (Bru et al., 2016; Solesio and Pavlov, 2016; Christ and Blank, 2018; Christ et al., 2020); the relatively low concentration of polyP in these organisms; and its dynamic nature, which makes the levels of polyP dependent on the specific cell type and the metabolic state of the cells.

To increase our knowledge of mammalian polyP and mitochondrial physiology, we have developed and characterized stable monoclonal SH-SY5Y cells that express PPX, PPN, and PPK enzymes in mitochondria, and PPX in the cytoplasm and endoplasmic reticulum (ER). To conduct our studies, we have optimized DAPI-based spectrophotometric assays to quantify polyP in mammalian cells. Our results corroborate the potent effects of polyP in the regulation of mammalian bioenergetics, and they show that polyP also affects gene expression and mitochondrial architecture. Furthermore, we are the first to demonstrate that the modification of the levels of cytoplasmic and ER polyP also has a deleterious effect on cellular bioenergetics and gene expression. Finally, we show the substantive influence of polyP chain length (in cells expressing PPN vs. PPX) in mammalian bioenergetics. Our results provide the research community with a toolkit of cellular models to study mammalian polyP and they increase our knowledge regarding its effects on mitochondrial physiology.

## Materials and methods

### Reagents

Dulbecco’s Modified Eagle medium (DMEM):F12, penicillin/streptomycin, Hank’s Balanced Salt Solution (HBSS), geneticin, trypsin, and heat-inactivated fetal bovine serum (FBS) were purchased from Gibco-Invitrogen (Carlsbad, California, US). 4′,6-diamino-2-phenylindole (DAPI), lipofectamine, alkaline phosphatase, Pierce BCA protein assay kit, Pierce ECL western blotting substrate, Pierce Halt protease and phosphatase Inhibitor Cocktails, Opti-MEM, ER-Tracker Red, and 0.1 M Cacodylate buffer were purchased from Thermo Fisher Scientific (Waltham, Massachusetts, US). Methanol, Phosphate-Buffered Saline (PBS), β-mercaptoethanol, tris(hydroxymethyl)-1,3-propanediol hydrochloride (TRIS-HCl), glycerol, phenylmethylsulfonyl fluoride (PMSF), Tween-20, Dimethyl sulfoxide (DMSO), potassium chloride, poly-L-lysine, [4-(2-hydroxyethyl)-1-piperazineethanesulfonic acid] (HEPES), sucrose, mannitol, ER-isolation kit, tetramethylrhodamine methyl ester perchlorate (TMRM), ethylenediaminetetraacetic acid (EDTA), ethylene glycol-bis (2-aminoethylether)-N,N,N’,N’-tetraacetic acid (EGTA), magnesium chloride, 1,4-dithiothreitol (DTT), Hoechst 33,342, Triton X-100, 25% glutaraldehyde, 4% paraformaldehyde, osmium tetroxide, acetone, epon resin, uranyl acetate, and lead citrate were obtained from Sigma Aldrich (St. Louis, Missouri, US). All the materials and reagents used in immunoblots, including secondary antibodies (anti-mouse, cat. num.: 1,706,516; anti-rabbit, cat. num.: 1,760,515), polyvinylidene (PVDF) membranes, fat-free milk, protein ladders, and polyacrylamide-precast gels were obtained from BioRad (Hercules, California, US). Anti-OXPHOS (ab110411), anti-PPX (ab225684), and anti-β-actin (ab8226) primary antibodies were obtained from Abcam (Cambridge, Cambridgeshire, United Kingdom). Anti-calreticulin (CST 2891S), and anti-TOMM20 (CST 42406S) antibodies were purchased from Cell Signaling Technology (Danvers, Massachusetts, US). Unless otherwise stated, all the reagents used on the Seahorse experiments were purchased from Agilent technologies (Santa Clara, California, US). scPPX enzyme was purchased from James H. Morrissey’s’ Laboratory (University of Michigan, Michigan, US). Short (≃ 14 Pi) chain synthetic polyP was a kind gift from Toshikazu Shiba (Kitasato University, Tokyo, Japan).

### Cell cultures

SH-SY5Y cells were purchased from the American Type Culture Collection (Manassas, Virginia, US) and maintained in culture following the provider’s instructions, and as we have previously done (Solesio et al., 2012; Solesio et al., 2013a; Angiulli et al., 2018; Solesio et al., 2018). Briefly, cells were grown using DMEM:F12 media supplemented with 10% (v/v) heat inactivated fetal bovine serum, and 10 units/mL of penicillin/streptomycin. To conduct our experiments, cells were grown to 80%–90% confluency in a humidified cell culture incubator (Heracell Vios 160i, Thermo Fisher Scientific, Waltham, Massachusetts, US), under a 5% CO_2_ atmosphere at 37°C. All the cells used for this project were grown and maintained under the same conditions.

### Generation of stable cell lines

SH-SY5Y cells were transfected with specific DNA constructs allowing for expression in mammalian cells of each of the main enzymes involved in the metabolism of polyP in microorganisms and lower eukaryotes (PPX, PPN, and PPK, sequence was obtained from *Saccharomyces cerevisae*). The vectors also contained a signaling sequence to express these proteins either in mitochondrial matrix, cytoplasm or ER; as well as the sequence for expression of GFP tag, in all the cases except CytoPPX. We also transfected SH-SY5Y cells with MitoGFP, a commercially available construct containing a mitochondrial targeting sequence and the sequence for expression of GFP.

Transfections were conducted following our previously established protocol (Guitart-Mampel et al., 2022; Hambardikar et al., 2022). Precisely, 0.3 × 10^6^ cells/well were plated in 6-well plates. 48 h later, cells were transfected using 800 µL of Opti-MEM, 12 µL of lipofectamine, and 3.2 µg of the respective DNA per well. 24 h post-transfection, cells were treated with 0.5 mg/mL of the selection antibiotic (geneticin). During the next 2 weeks, medium was replaced by fresh medium containing geneticin every 2 days. Subsequently, transfected cells were diluted in 96-well plates and grown further. Single cell colonies were then selected for amplification by visualization of GFP, using EVOS AMF4300 microscope (ThermoFisher Scientific, Waltham, Massachusetts, US). Cell colonies that expressed low or no GFP signal were discarded. CytoPPX cells lack the GFP marker due to the design of the construct, hence the imaging step was skipped for these colonies. Instead, the final cells were selected on the basis of the presence of the PPX protein in the cytoplasm of the transfected monoclonal stable cells.

### Confocal and fluorescence microscopy

To confirm the co-localization of GFP and mitochondria or ER in the newly created SH-SY5Y cells, we labeled each of the organelles using specific fluorescence probes. Specifically, mitochondria were labeled with TMRM, following the protocol that we previously used (Solesio et al., 2021); while the ER was labeled using ER-tracker Red, following the instructions provided by the manufacturer. Live cell imaging was then performed using confocal microscopy (Leica SP8, Wetzlar, and Germany), with a ×40 oil immersion objective.

Wild-type (Wt) cells that were transfected with the MitoPPK or the MitoGFP constructs, or non-transfected were monitored over time using fluorescent microscopy. Specifically, 24 h after transfection, cells were imaged using an EVOS AMF4300 microscope (ThermoFisher Scientific, Waltham, Massachusetts, US) to detect GFP signal and confirm successful transfection and expression of the specific plasmids. Subsequently, geneticin was added to the growing medium. Seven days post-transfection, cells were stained with DAPI and incubated for 15 min at 37°C in the dark. Cells were then imaged again using transmitted light; the DAPI and GFP filters; and the same microscope.

### Cell fractioning

Mitochondrial fractions from MitoPPX cells were isolated following the protocol that we previously used (Solesio et al., 2018; Hambardikar et al., 2022). Cytoplasmic fractions from CytoPPX cells, and mitochondrial fractions from CytoPPX and MitoPPN cells were collected from 150 mm petri plates. Specifically, cells were scraped and resuspended in fractionation buffer (20 mM HEPES, 10 mM KCl, 2 mM MgCl_2_, 1 mM EDTA, 1 mM EGTA, 1 mM DTT and 1X protease inhibitor), and incubated for 15 min on ice. Subsequently, they were passed through a 20-gauge needle 40 times. After a 20 min incubation on ice, cells were centrifuged at 720 x*g* (Eppendorf 5430 R, Hamburg, Germany) for 5 min at 4°C. The collected supernatants were centrifuged at 10,000 x*g* for 5 min at 4°C, using the same centrifuge. The obtained supernatants contained the cytoplasmic fractions, while the pellets contained the mitochondrial fractions. Lastly, ER fractions were collected using the ER isolation kit, following the manufacturers’ protocol. For final last step, we obtained a pellet containing rough ER-enriched microsomes, which was used for our experiments.

Lysis and protein quantification of the collected fractions were performed on the day of the experiments. The mitochondrial and ER fractions were re-suspended in 25–30 µL of lysis buffer. All the fractions (mitochondrial, ER, and cytoplasm) were then lysed by three freeze/thaw cycles, followed by sonication at 30% amplitude (QSONICA sonicator, Newtown, Connecticut, US), and centrifugation at 18,000 x*g* for 5 min at 4°C (Eppendorf 5430R, Hamburg, Germany). Protein was quantified on the obtained supernatants (lysates) using the Pierce BCA protein assay kit, following the protocol provided by the manufacturer.

### Immunoblots

Immunoblots were conducted following the previously described protocol (Solesio et al., 2012; Solesio et al., 2013a; Baltanas et al., 2013), and using 10 ug of protein per condition. Protein quantification was conducted as indicated above. All primary and secondary antibodies were used at 1:1,000 dilution. The signal was detected using the Gel Doc XR Image System form BioRad (Hercules, California, US). TOMM20 protein levels were used to confirm mitochondrial enrichment in the mitochondrial fractions, calreticulin levels to confirm ER enrichment in the ER fractions, and β-actin levels as loading controls in the rest of the experiments.

### Cell fixation and EM imaging and quantification

2 × 10^6^ cells were trypsinized and spun down (Eppendorf Centrifuge 5910R, Hamburg, Germany) at 1,200 x*g* to form a pellet. Cellular pellets were then fixed using a solution composed by 2.5% glutaraldehyde and 4% paraformaldehyde in 0.1 M cacodylate buffer. Subsequently, they were post-fixed in buffered 1% osmium tetroxide, dehydrated in a graded series of acetone, and embedded in epon resin. 90 nm-thin sections were cut using a Leica EM UC6 ultramicrotome (Leica, Heidelberg, Germany). Sectioned grids were stained with a saturated solution of uranyl acetate and lead citrate. Images were captured using an AMT XR111 digital camera (McLean, Virginia, US) on a Philips CM12 transmission electron microscope (Amsterdam, Netherlands).

Mitochondria were quantified in the obtained images using a blind method (the images were unidentified for the counter), following a similar protocol as we did in the past (Solesio et al., 2012; Solesio et al., 2013a; Solesio et al., 2013b). Mitochondria were grouped into normal, electron lucent and electron dense. Four images per cells type were quantified and a percentage of each group of mitochondria was determined based on total mitochondria in each image.

### DAPI-polyP spectrophotometric assay

The DAPI-polyP complex shifts the fluorescence emission of DAPI to 550 nm, which allows for the visualization of polyP using microscopy (Aschar-Sobbi et al., 2008). Subcellular fractions from Wt cells were used as control conditions in these experiments. 50 μL of lysates from various subcellular fractions were loaded into 96-well black half-area plates, in triplicate and at a protein of concentration of 0.1 μg/μL. PolyP standards (0, 2.5, 5, and 10 µM) were also prepared, using short chain synthetic polyP. PolyP standards and samples were diluted in a buffer containing 75 mM sucrose, 225 mM mannitol, and 5 mM Tris-HCl. DAPI was added to each well at a final concentration of 10 µM. Fluorescence was then quantified after 30 min dark adaptation at 37°C, using a BioTek spectrophotometer (Thermo Fisher Scientific, Waltham, Massachusetts, US), and a λ_ex_ = 415 nm and λ_em_ = 550 nm. DAPI signal for each sample was corrected for background and the values were normalized to the signal obtained from the Wt samples.

To validate the spectrophotometric assay based on DAPI-polyP fluorescence, we quantified DAPI fluorescence at 550 nm in i) isolated mitochondria from Wt cells and, 2) 10 μM short chain polyP, in the presence or absence of PPX and Alkaline Phosphatase (AP), two main enzymes involved in the degradation of polyP. Samples were incubated for 15 min at 37°C with 5 μg/mL of PPX enzyme or 5 U/µL of AP in the presence of 15 mM of MgCl_2_ as a cofactor. DAPI-polyP fluorescence was then assayed as previously described.

### Densitograms and pearson’s coefficients

Densitograms to determine colocalization between TMRM or ER-tracker (indicating the specific subcellular organelles), and GFP (indicating PPX) were performed using the RGB profile plot plugin from ImageJ (NIH, Bethesda, MD, United States), as indicated in the images. Using the same software, Pearson’s coefficients were determined using JaCOP (Just another colocalization plugin).

### PPX and PPN enzymatic activity

The activity of the polyP hydrolyzing enzymes, PPX and PPN, were assayed by the quantification of the degradation of short chain exogenous polyP in cell lysates. While the length of mammalian polyP still remains controversial; it has been proposed that this polymer is usually found in two pools in cell lines: very long chains, and short lengths (Kumble and Kornberg, 1995). We decided to use short chains of polyP to conduct all our studies because, based on the bibliography, long chains of the polymer induce major effects in the transcriptome, proteome, and phosphatome of mammalian cells (Bondy-Chorney et al., 2020; Pirttiniemi et al., 2023). Moreover, the data included in this manuscript show that the expression of MitoPPK is lethal for SH-SY5Y cells.

To conduct these experiments, we used a modified version of the protocol previously established by our laboratory (Solesio et al., 2021). Specifically, cells were collected and lysed, and protein levels were quantified as described above. Samples were then diluted in incubation buffer (0.1% BSA (w/v), 10% glycerol, 20 mM TRIS-HCl (pH = 7.5), and 50 mM potassium chloride) to 0.25 μg/μL in 20 µL. This buffer was used for all the solutions in this assay. A solution containing 5 mM of short chain polyP was also prepared to be used as the substrate of the enzymes. scPPX1 was used as a positive control for these experiments, at a final concentration of 5 μg/μL in 20 µL. DAPI was dissolved to 40 μg/μL and 99 μL of this solution were added to each well of a 96-well black (clear bottom) plate. To initiate the enzymatic reaction, 20 μL of polyP (substrate) were added and mixed with 20 μL of the cell lysates in a 1.5 mL tube. Immediately after that, 1 μL of the reaction mix was added to each well. Each sample was added in quadruplicates. Fluorescence was measured over time, at 550 nm. Results were analyzed after background correction and normalized to Wt florescence at each time point.

### Seahorse assays

The status of OXPHOS was assessed using a Seahorse XFe24 Analyzer (Agilent, Santa Clara, California, US). Briefly, 4 × 10^4^ cells/well were seeded in an Agilent Seahorse XF24 cell culture microplate, using high glucose DMEM. Subsequently, cells were incubated overnight under standard conditions. The medium was then replaced by Agilent XF medium (DMEM containing 5 mM HEPES without phenol red, pH = 7.4) supplemented with 10 mM glucose, 1 mM pyruvate, and 2 mM L-glutamine. The Mito Stress test was preceded by initial incubation of the cells at 37°C without CO_2_ for at least 45 min, to ensure the pre-equilibration of the assay medium. The assay was carried out by first measuring baseline oxygen consumption rate (OCR), followed by sequential measurements of OCR and extracellular acidification rate (ECAR) after the injection of 1 µM oligomycin, 2 µM FCCP, and 0.5 µM rotenone + antimycin A. Specifically, basal respiration represents the OCR before the addition of any of the drugs; proton leak the OCR after the addition of oligomycin; and ATP-linked respiration represents the value of the OCR resulting from the subtraction of the proton leak from the basal respiration. Moreover, maximal respiration, represents the value that the OCR reached after the addition of FCCP; while the non-mitochondrial respiration, is the OCR assayed after the addition of rotenone + antimycin A. Finally, the spare capacity was calculated by subtracting the basal respiration from the maximal respiration. All these calculations were conducted following the manufacturer’s instructions.

Cell count-based normalization of real-time data was obtained by staining cells with 5 µM Hoechst 33,342 and counting the cells on the BioTek Cytation 1 Imaging Multi-Mode Reader (Agilent, Santa Clara, California US).

### Gene expression

#### RNA isolation

Total RNA was isolated from approximately 1 × 10^6^ cells, using the RNeasy Micro Kit (QIAGEN, Hilden, Germany), and following the manufacturer’s protocol. Total RNA concentration and integrity were estimated by mass spectrophotometry (NanoDrop One, GE Health Care, Buckinghamshire, United Kingdom).

#### cDNA synthesis

cDNA was synthetized from 1 µg of total RNA using the QuantiTect Rev. Transcription Kit (QIAGEN, Hilden, Germany) following the manufacturer’s instructions.

#### RT-qPCR

Specific primer sets for each gene were designed using Primer3web 4.1.0 (available at https://primer3.ut.ee/). The expression of the housekeeping gene GAPDH (glyceraldehyde 3-phosphate dehydrogenase) was used to normalize the relative expression of target genes. Primer sequenced and amplicon sizes are shown below. Amplifications were performed in a QuantStudio™ 6 Flex Real-Time PCR System (Applied Biosystems, Foster City, California, US), in a final volume of 10 µL using the fluorescent dye Power SYBR™ Green PCR Master Mix (QIAGEN, Hilden, Germany) and 25 µM of each primer. The thermal conditions were an initial hot start step at 95°C for 10 min, followed by 40 cycles of 95°C for 15 s, and 60°C for 25 s. Differential gene expression between groups were accessed by the 2^−ΔΔCq^ method (Livak and Schmittgen, 2001). The results are shown in fold change values.

### Primers used

**Table:** 

Gene	Forward and reverse sequences	Amplicon (bp)
GAPDH	TTG​GCT​ACA​GCA​ACA​GGG​TG	161
	GGG​GAG​ATT​CAG​TGT​GGT​GG	
SOD2	TGG​GGT​TGG​CTT​GGT​TTC​AA	95
	GGA​ATA​AGG​CCT​GTT​GTT​CCT​TG	
SIRT3	CGG​CTC​TAC​ACG​CAG​AAC​ATC	225
	CAG​CGG​CTC​CCC​AAA​GAA​CAC	
MFN2	ATG​TCC​CTG​CTC​TTC​TCT​CG	202
	GGT​CCA​GTT​CTG​CAT​TCC​TG	
TOMM20	CCC​CAA​CTT​CAA​GAA​CAG​GC	185
	GAT​GGT​CTA​CGC​CCT​TCT​CA	
DNM1L	AGA​ATA​TTC​AAG​ACA​GTG​TGC​CA	145
	TGT​GCC​ATG​TCC​TCA​GAT​TCT	
PRKN	GTG​CCG​TAT​TTG​AAG​CCT​CA	123
	GAC​AGG​GCT​TGG​TGG​TTT​TC	

### Statistical analysis

The qPCR data were analyzed in fold change values, *n* = 4-5 biological replicates. Comparisons between multiple groups were performed with one-way ANOVA with Tukey’s *post hoc* analyses. PolyP quantification and Seahorse assays were conducted in biological triplicates (*n* = 3). The immunoblots to determine the presence of the studied enzymes were not conducted in triplicate, due to the nature of the samples. Statistical significance of the differences between groups in these experiments was determined by Student’s t-test, one-way ANOVA (Turkey *post hoc* test).

All data is presented as mean ± SEM. The level of statistical significance was set at α = 0.05 (*p ≤ 0.05, **p ≤ 0.01, ***p ≤ 0.001). For the statistical analysis and graphic representation, OriginLab software and GraphPad Prism version 9 were used. (Northampton, MA, US; and San Diego, CA, US; respectively).

## Results

### Biochemical characterization of the cellular models

**FIGURE 1:**
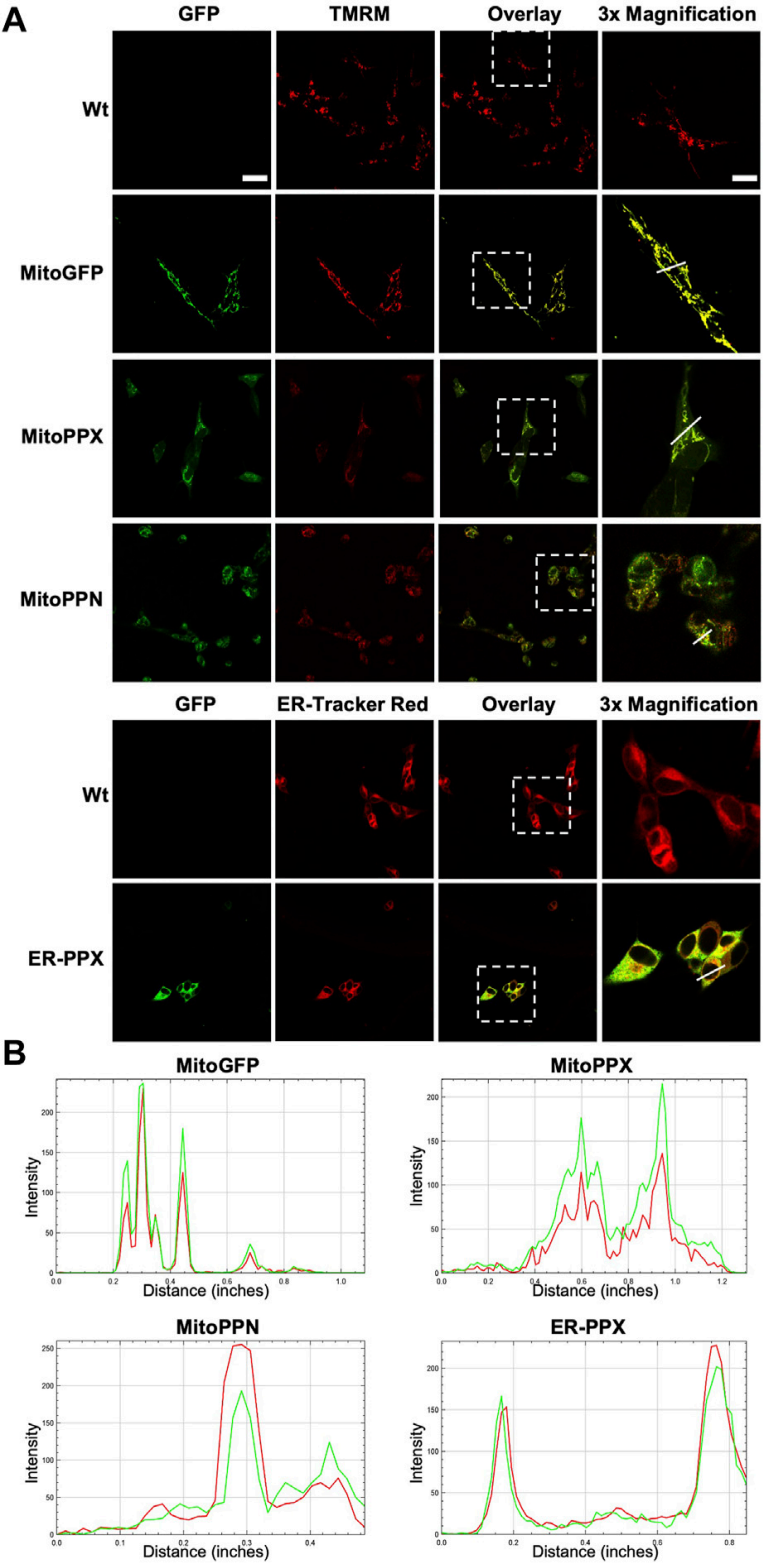
*Confocal microscopy images confirm the mitochondrial expression of GFP in mitochondria from MitoPPX and MitoPPN SH-SY5Y cells, as well as the expression of GFP in ER from ER-PPX SH-SY5Y cells.* **(A)**. Representative confocal images of Wt, MitoGFP, MitoPPX, MitoPPN, and ER-PPX cells. ER was labeled using ER-Tracker Red, while mitochondria were labeled using TMRM. Images that show the overlap of the GFP signal and ER-Tracker Red or TMRM are included. Magnifications (×3) of significant areas obtained from the overlay images are also included. Scale bar = 50 µm for all the images except for the magnifications. In that case, scale bar = 17 µm. **(B)**. Densitometry conducted along the lines that are marked in the magnification images included in Figure 1A. Green: GFP, Red: specific markers of mitochondria or ER.

**FIGURE 2:**
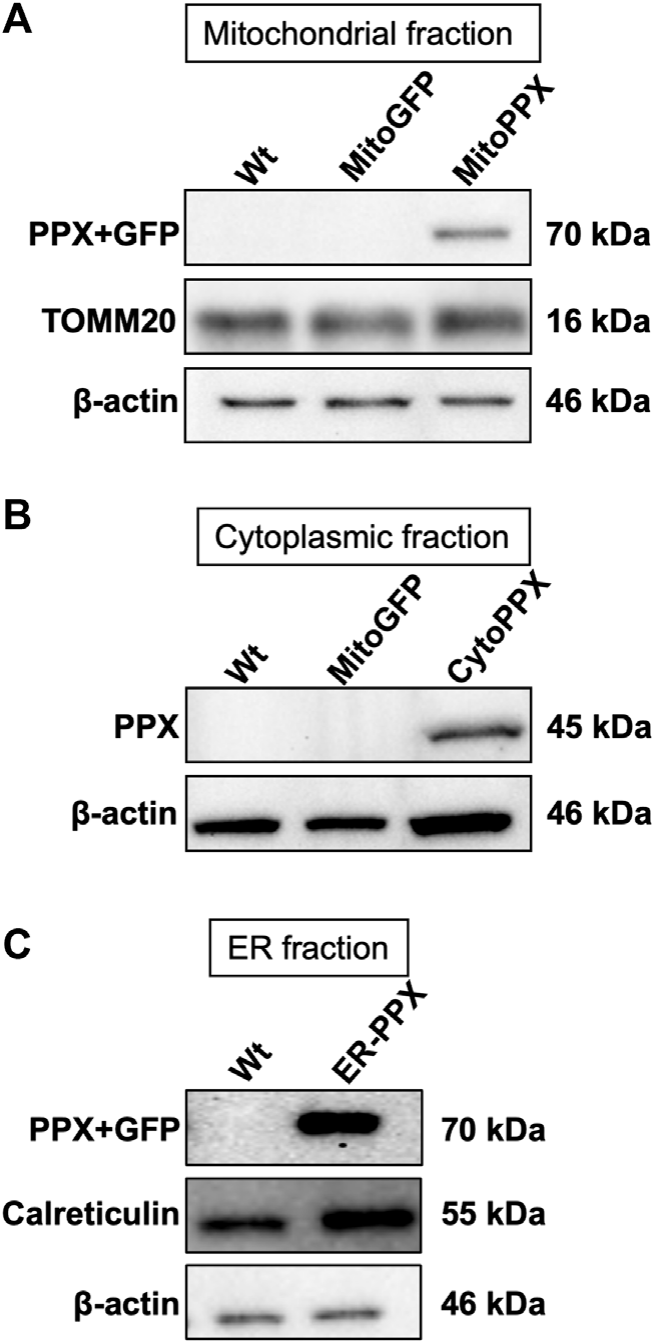
*PPX is expressed in mitochondria from MitoPPX, cytoplasm from CytoPPX, and ER from ER-PPX SH-SY5Y cells.* **(A)**. Representative immunoblots showing the presence of the PPX-GFP complex (70 kDa) in the mitochondrial fraction of MitoPPX cells, as well as the absence of the same complex in the mitochondrial fractions of Wt and MitoGFP SH-SY5Y cells. Please note that the levels of GFP were not assayed in this figure. TOMM20 levels indicate mitochondrial enrichment in the assayed fractions; while low β-actin levels indicate decreased presence of cytoplasmic proteins in the mitochondrial fractions. MitoPPN cells were not assayed because no antibodies for the PPN protein are available. **(B)**. Representative immunoblots that show the presence of the PPX protein (45 kDa) in the cytoplasmic fractions of CytoPPX cells, and its absence in the corresponding fractions of Wt and MitoGFP SH-SY5Y cells. Note that the CytoPPX construct does not contain the sequence for the expression of GFP. β-actin signal indicates cytoplasmic enrichment in the assayed fractions. **(C)**. Representative immunoblots that show the presence of the PPX-GFP protein complex (70 kDa) in the ER fraction of ER-PPX SH-SY5Y cells, as well as the absence of the same complex in the ER fraction of the Wt cells. Calreticulin presence indicates ER enrichment in the assayed fractions, and low β-actin signal indicates decreased presence of cytoplasmic proteins.

Confocal imaging was performed to ensure colocalization between GFP-PPX and GFP-PPN; and mitochondria or ER (Figure 1A). The CytoPPX plasmid did not express GFP, therefore CytoPPX cells were not included in these experiments. Densitogram of representative areas included in the magnification images (Figure 1B), as well calculation of the Pearson’s coefficient (Table 1), corroborated the specific presence of PPX in the different subcellular compartments, along with its co-localization with these compartments. The results were further validated by western blot analysis of subcellular fractions, which showed the presence of PPX in the appropriate fractions (Figure 2). Due to the lack of availability of antibodies against PPN, immunoblots were not performed for detection of these protein in MitoPPN cells. However, GFP signal (a GFP tag is present in the MitoPPN construct) was visualized using confocal microscopy imaging (Figure 1). It was not possible to detect GFP protein in mitochondrial fractions from MitoPPN cells by immunoblotting, despite GFP expression. This discrepancy could be explaining by conformational changes in the GFP protein, not allowing for the recognition by the specific epitope of the antibody.

**TABLE 1:** *The specific proteins expressed in the various subcellular locations co-localize with the markers used for visualizing these locations.* Person’s coefficients were calculated for all the conditions included in Figure 1. Note the significant co-localization observed in MitoGFP, MitoPPX, MitoPPN, and ER-PPX cells (Pearson’s coefficient higher than 0.5 and close to 1) indicating the connection between the PPX or the PPN protein and mitochondria or the ER. Image A: red channel, image B: green channel.

Cell type	Pearson’s Coefficient (*r* =)
MitoGFP	0.988
MitoPPX	0.957
MitoPPN	0.97
ER-PPX	0.95

### Mitochondrial expression of PPX and PPN induces alterations of mitochondrial architecture

**FIGURE 3:**
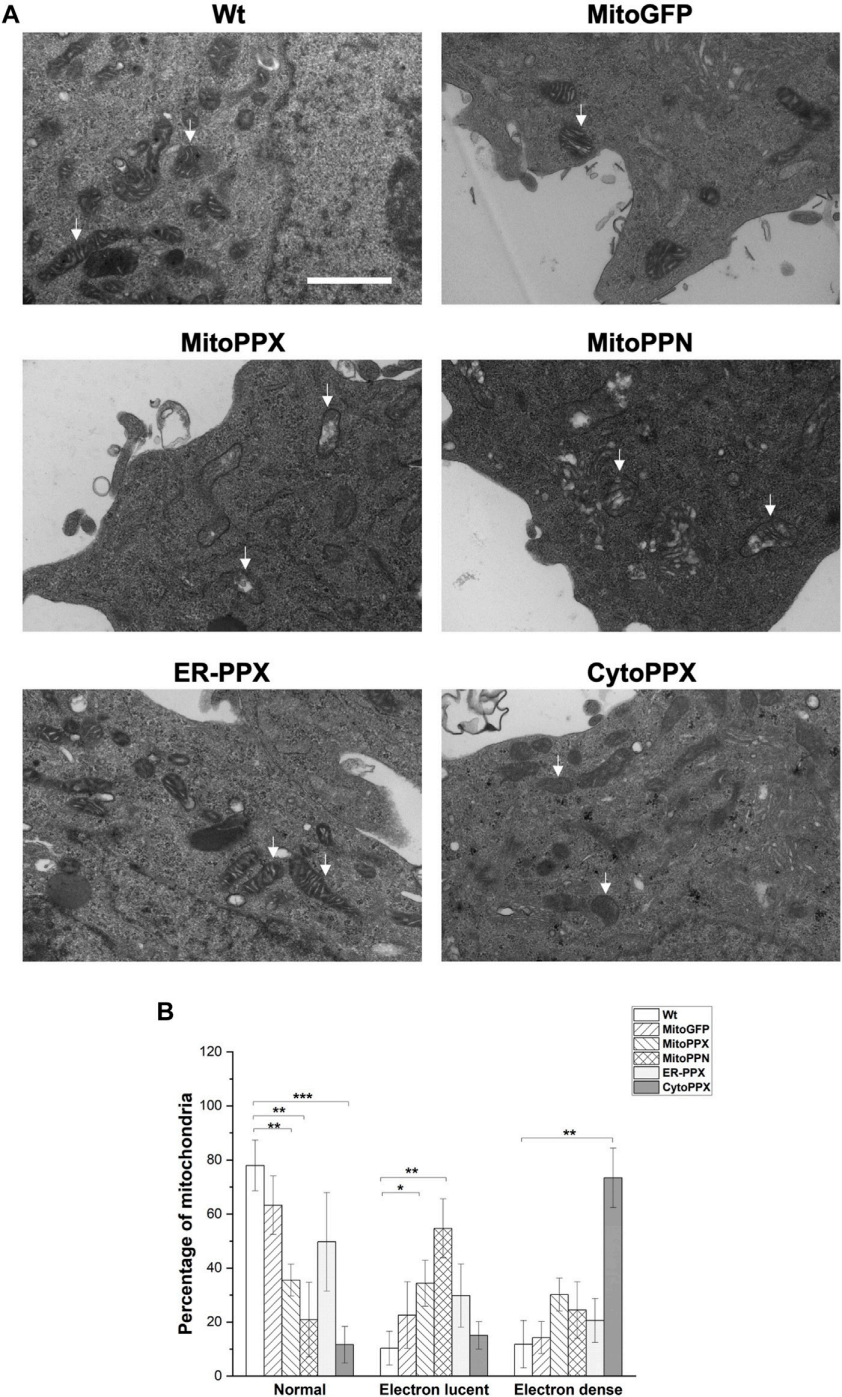
*Depletion of mammalian polyP causes structural mitochondrial changes*. **(A)**. Representative images of Wt, MitoGFP, MitoPPX, MitoPPN, ER-PPX and CytoPPX cells. These images were obtained using EM. Arrows point towards significant mitochondria. Scale bar = 500 nm. **(B)**. Quantification of the images. To conduct this quantification, mitochondria from all the conditions were classified as normal, electron lucent, and electron dense. MitoPPX and MitoPPN cells showed a clear increase in the number of electron lucent mitochondria, while CytoPPX cells showed increased electron dense mitochondria. Graphs represent average ±SEM of four independent images. Statistical analysis conducted by unpaired *t*-test with α = 0.05 (* *p* ≤ 0.05, ** *p* ≤ 0.01, *** *p* ≤ 0.001).

Using Electron Microscopy (EM), we imaged all the cell lines that we have created (Figure 3A). This allowed us to visualize mitochondrial ultrastructural changes in response to the modification of the levels of polyP in the different subcellular compartments. Wt and MitoGFP cells were used as controls, as mitochondria from these cells showed the standard shape, including an intact inner membrane and well-defined cristae projecting into the matrix. MitoPPX mitochondria showed increased presence of electron lucent mitochondria, and this effect was of a larger magnitude in mitochondria from MitoPPN cells. Moreover, mitochondria from ER-PPX cells showed a morphology similar to the Wt samples. In fact, quantification of these images (Figure 3B), showed that, compared to Wt cells, MitoPPX samples had 42.4% less normal mitochondria and 24.1% more electron lucent mitochondria; while MitoPPN cells showed 57% less normal mitochondria and 44.4% higher electron lucent mitochondria. CytoPPX cells showed lower percentage of normal mitochondria, 66.3% less than Wt. Moreover, they had the highest presence of electron dense mitochondria (61.6% more than Wt cells). ER-PPX cells showed no significant alterations in mitochondrial structure. Based on all the data that we obtained, we concluded that Wt and MitoGFP cells have similar mitochondrial morphology, without any differences in terms of electron density. Therefore, we used Wt cells as control samples for the rest of the experiments included in this manuscript.

### Targeted expression of polyP metabolizing enzymes modulates polyP levels in the specific subcellular compartments

**FIGURE 4:**
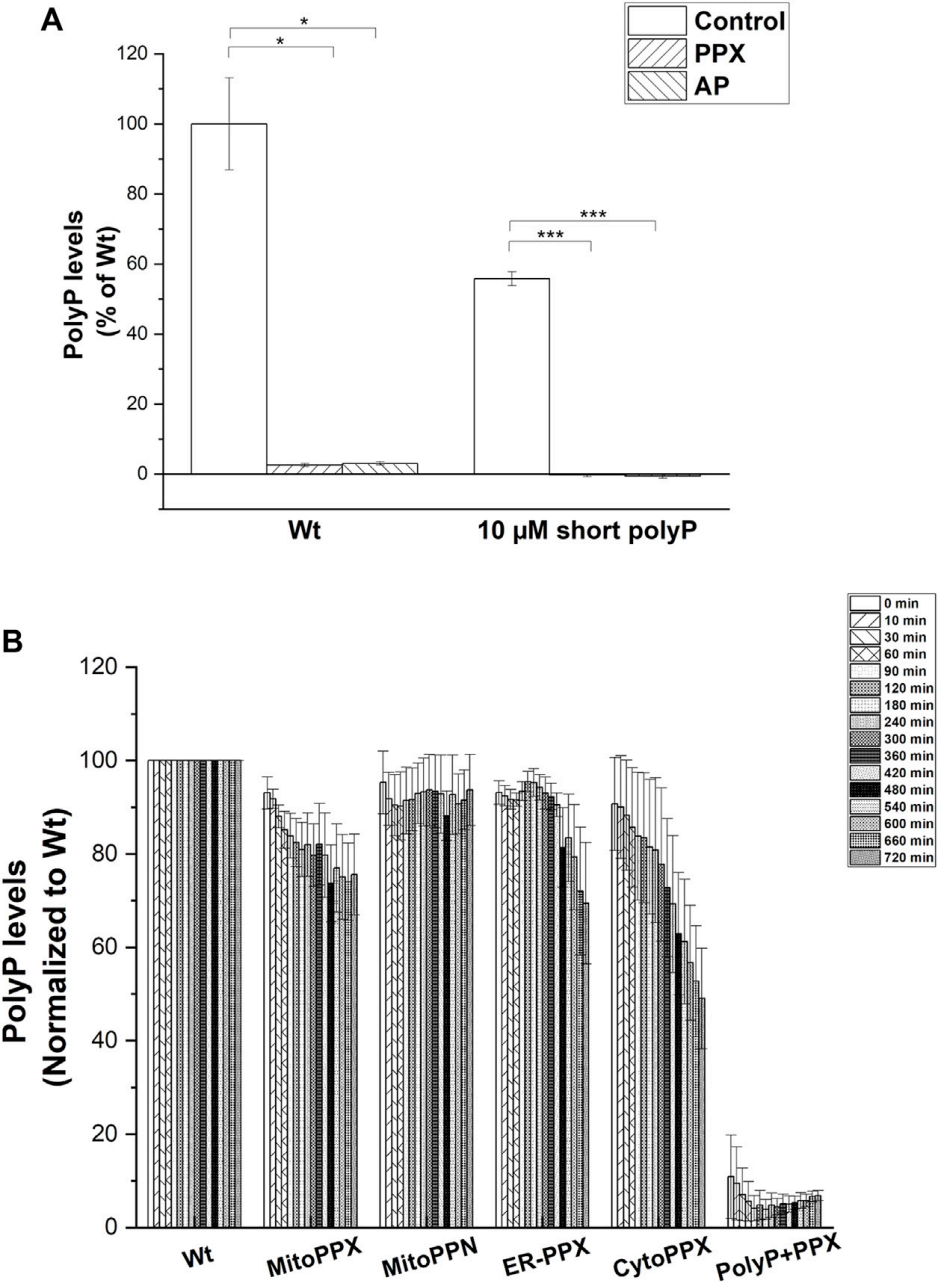
**(A)**. *PPX and PPN enzymes are active in mutant SH-SY5Y cells.* **(A)**. Graph showing the kinetics of the PPX and PPN enzymes in the different subcellular compartments in SH-SY5Y cells. To conduct these studies, we treated the cells with exogenous PPX and AP. Subsequently, we assayed DAPI-polyP fluorescence. Note the sharp decrease in the fluorescence levels of DAPI-polyP after treatment with PPX and AP, in both mitochondria isolated from Wt cells and exogenous polyP. Graphs represent average ±SEM of three independent experiments. Statistical analysis was conducted by unpaired *t*-test. α = 0.05 (* *p* ≤ 0.05, *** *p* ≤ 0.001). **(B)**. Cell lysates obtained from Wt, MitoPPX, MitoPPN, ER-PPX, and CytoPPX were incubated with exogenous, short chain polyP (concentration is expressed in terms of Pi) and DAPI. Enzymatic activity was assayed by quantification of DAPI-polyP fluorescence over 12 h. PolyP treated with the scPPX enzyme was used as control. Measurements were normalized to the Wt signal at each time point. Graph shows representative enzymatic activity assay.

Subsequently, we assayed the enzymatic activity of the polyP hydrolyzing enzymes. To do this, exogenous polyP was added to the cell lysates and its degradation was assayed by measuring DAPI-polyP fluorescence (Abramov et al., 2007). Treatment with exogenous PPX was used to demonstrate the specificity of the DAPI-polyP binding, and alkaline phosphatase (AP) was used as a further positive control (Figure 4A). Specifically, MitoPPX, MitoPPN, CytoPPX and ER-PPX SH-SY5Y cells showed a time-dependent decrease of DAPI fluorescence, which indicated degradation of polyP. Wt cells, and polyP incubated with scPPX1 were used as controls for these experiments (Figure 4B).

**FIGURE 5:**
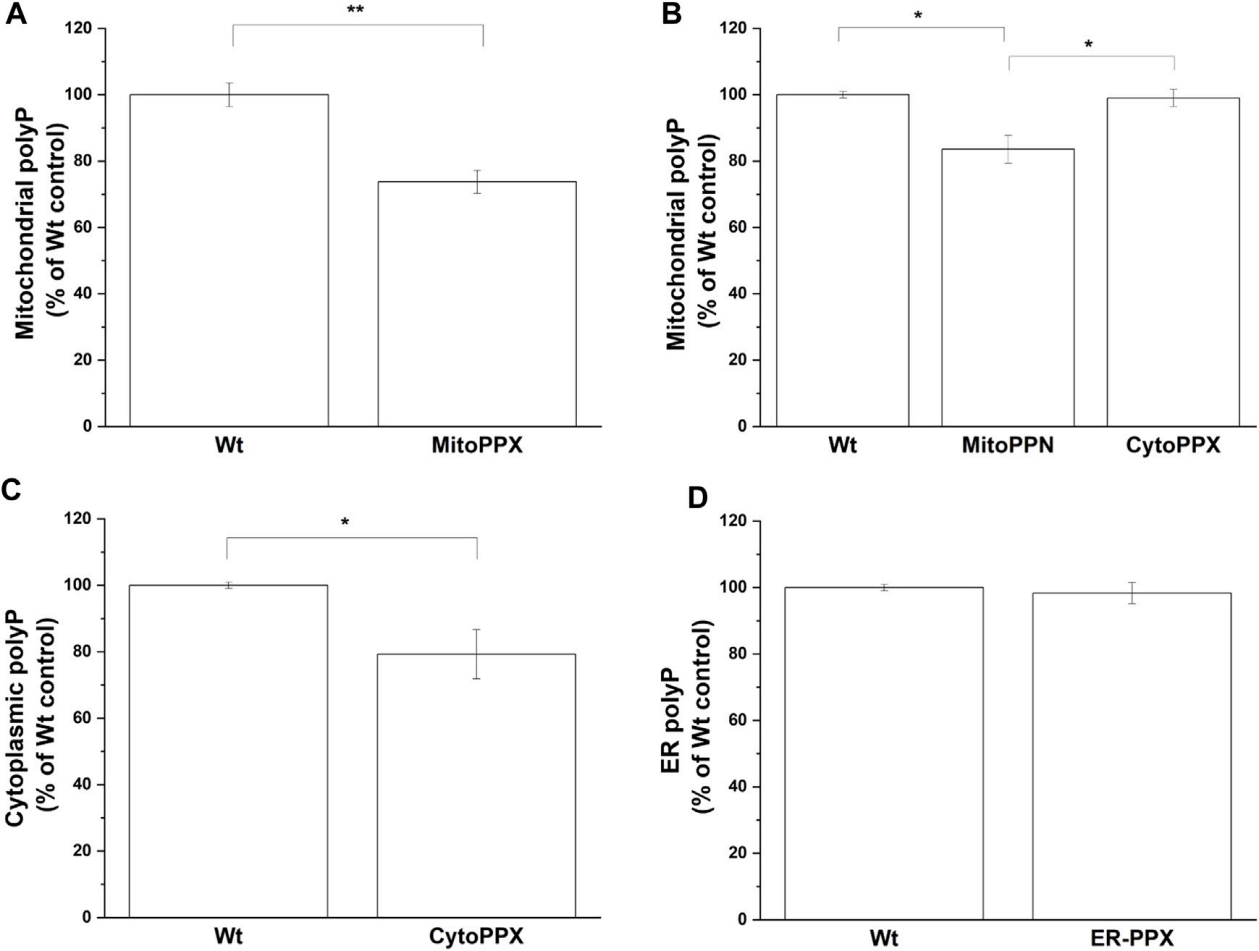
*The effects of PPX in SH-SY5Y cells are specific to the subcellular location where the enzyme is expressed.* By measuring DAPI-polyP fluorescence, we assayed the levels of polyP in mitochondrial fraction from **(A)**. MitoPPX, and **(B)**. MitoPPN and CytoPPX SH-SY5Y cells. Using the same method, polyP levels were also assayed in **(C)**. Cytoplasmic fraction from CytoPPX, and **(D)**. the ER fraction from ER-PPX cells. Corresponding Wt fractions are used as control in each of the experiments, and polyP levels were normalized to the values obtained in Wt cells. Graphs represent average ±SEM of three independent experiments. Statistical analysis was conducted by unpaired *t*-test. α = 0.05 (* *p* ≤ 0.05, ** *p* ≤ 0.01, *** *p* ≤ 0.001).

The levels of polyP in the different subcellular compartments were analyzed using an end-point DAPI-polyP spectrophotometric assay. Mitochondrial-enriched fractions from MitoPPX, MitoPPN and CytoPPX cells; cytoplasmic-enriched fractions from CytoPPX; and ER-enriched fractions from ER-PPX cells were isolated. Corresponding subcellular fractions from Wt cells were also isolated and used as controls for these experiments. Mitochondrial polyP levels showed a 25% decrease in MitoPPX cells, and a 16% decrease in MitoPPN cells, when compared to Wt cells (Figures 5A, B). CytoPPX cells showed 20.7% lower levels of polyP in their cytoplasmic fractions, when compared to Wt cells; while their levels of mitochondrial polyP were comparable to those found in the mitochondrial fractions of Wt cells (Figures 5B, C). ER-PPX cells did not show significant changes in polyP levels in the ER fraction, when compared to the ER fractions obtained for the Wt cells (Figure 5D).

### Modulation of polyP in different subcellular compartments causes differential gene expression

**FIGURE 6:**
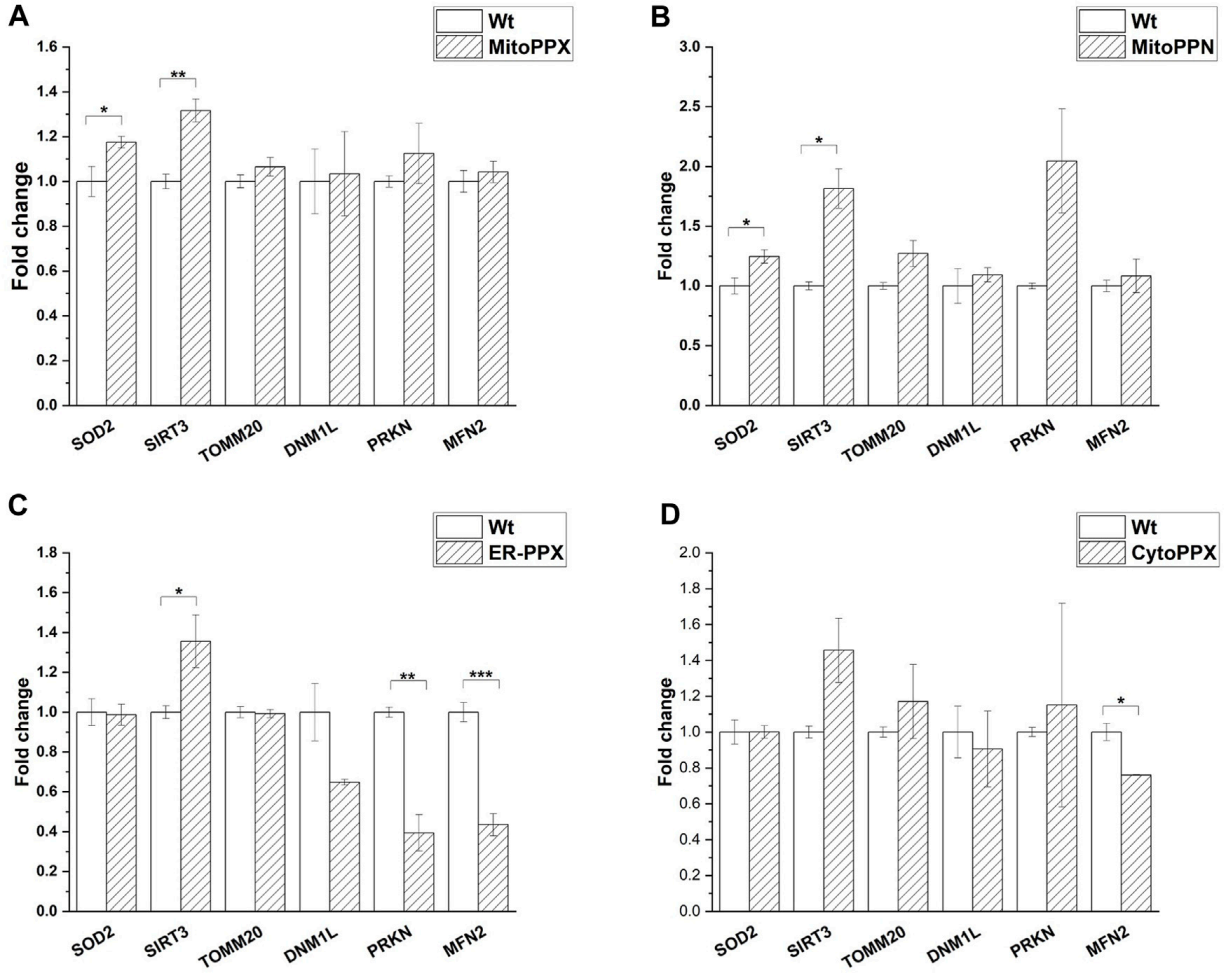
*Targeted expression of PPX in SH-SY5Y cells affects gene expression*. Expression of some of the main genes involved in mitochondrial physiology was assayed in all mutant cells. Expression levels of mutant cells were normalized with the values obtained in the Wt samples. **(A)**. MitoPPX, and **(B)**. MitoPPN cells showed increased expression of SOD2 and SIRT3. **(C)**. ER-PPX cells showed increased expression of SIRT3 and decreased expression of MFN2 and PRKN. **(D)**. CytoPPX cells showed decreased expression of MFN2. Graphs represent average ±SEM of three independent experiments. Statistical analysis was conducted by one-way ANOVA with Tukey’s *post hoc* analyses. α = 0.05 (* *p* ≤ 0.05, ** *p* ≤ 0.01, *** *p* ≤ 0.001).

mRNA fold change was quantified by qPCR for mitochondrial physiology related genes. Specifically, MFN2 (mitochondrial fusion), DNM1L (mitochondrial fission), PRKN (mitophagy), SOD2 (antioxidant system), SIRT3 (mitochondrial acetylation/deacetylation), and TOMM20 (mitochondrial content) expression were assayed. The obtained values were normalized against the housekeeping gene GAPDH (Figure 6). Our data show that SIRT3 was overexpressed in MitoPPX, MitoPPN and ER-PPX cells (1.31-, 1.81-, 1.35-fold change, respectively when compared to Wt cells). Moreover, in CytoPPX cells, even if not significant, the same tendency was observed. Furthermore, MitoPPX and MitoPPN cells showed increased expression of SOD2 (1.17-, and 1.24-fold change, respectively when compared to Wt cells) which remained unaltered in CytoPPX and ER-PPX cells. PRKN expression was decreased by 0.39-fold change in ER-PPX cells, while it showed an increased tendency in MitoPPN cells, even if this tendency was not significant. The expression of this gene remained unaltered in MitoPPX and CytoPPX cells. MFN2 expression was decreased in CytoPPX and ER-PPX cells by 0.76-, and 0.43-fold change, respectively; when compared to Wt samples. TOMM20 and DNM1L expression remained unaltered in all four cell types, despite altered polyP levels.

### Modulation of polyP in different subcellular compartments alters OXPHOS

**FIGURE 7:**
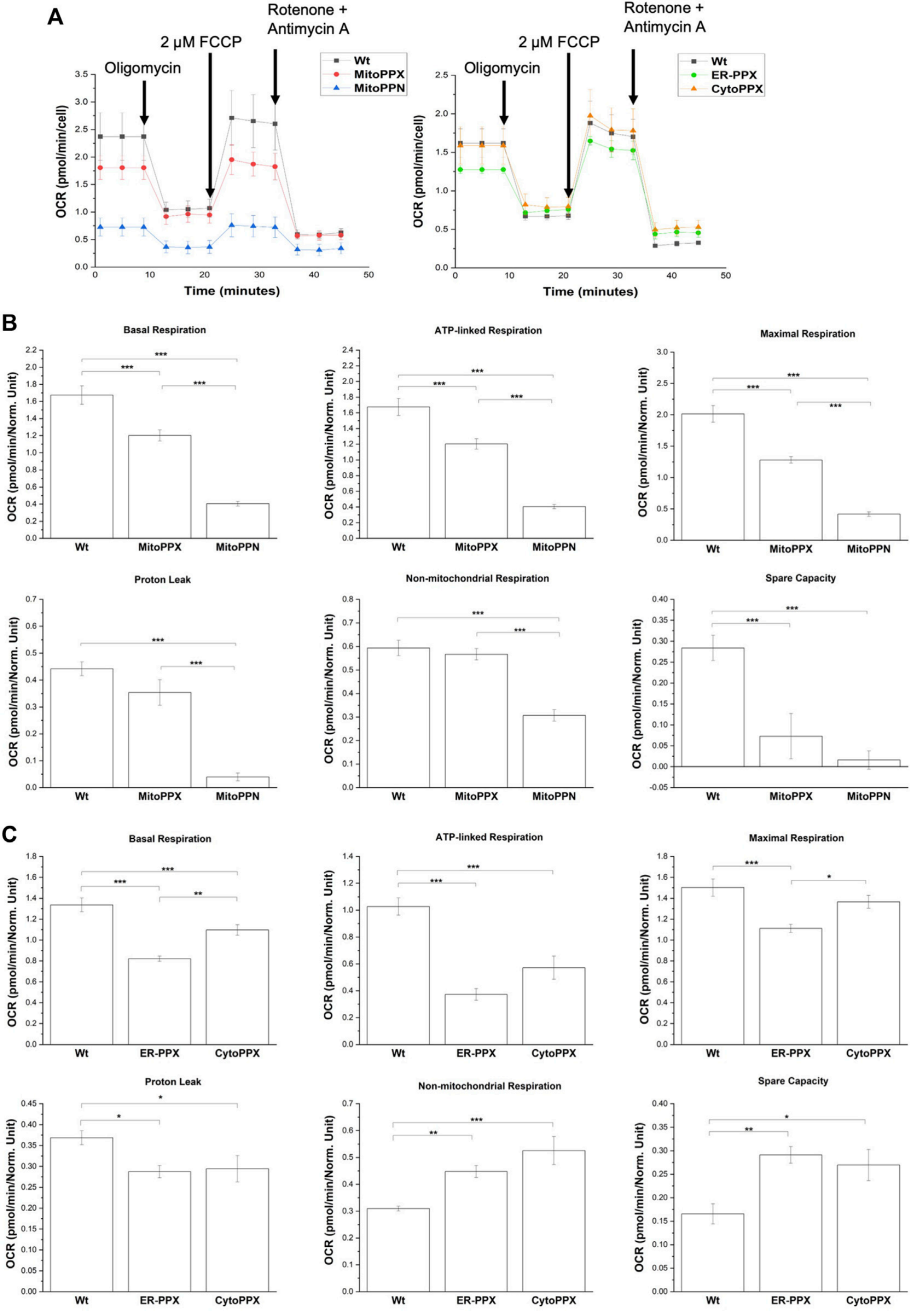
*Decreased levels of polyP affect the status of OXPHOS in SH-SY5Y cells.* **(A)**. Seahorse measurements that show OCR profiles in MitoPPN, MitoPPX, ER-PPX, and CytoPPX SH-SY5Y cells. Quantification of the Seahorse results showed the effects of the depletion of polyP in OCR in basal respiration, ATP-linked respiration, maximal respiration, proton leak, non-mitochondrial respiration, and spare capacity in **(B)**. MitoPPX and MitoPPN SH-SY5Y cells, and **(C)**. ER-PPX and CytoPPX cells. Wt cells were used as control in all the cases. Note that the depletion of mitochondrial polyP decreased OCR in all the cases; when the enzyme was expressed in ER or cytoplasm, this effect was less dramatic and, in some cases, even opposite. Graphs represent average ±SEM of three independent experiments. Statistical analysis conducted by one-way ANOVA and Tukey’s *post hoc* with α = 0.05 (* *p* ≤ 0.05, ** *p* ≤ 0.01, *** *p* ≤ 0.001).

Mitochondrial respiration was assayed using a Seahorse analyzer (Figure 7). Basal respiration and ATP-linked respiration were significantly decreased in all four cell lines, when compared to the Wt samples. Specifically, when compared to the Wt samples, basal respiration was decreased by 30% in MitoPPX, 77% in MitoPPN, 38% in ER-PPX, and 18% in CytoPPX cells. Moreover, ATP-linked respiration was decreased by 33% in MitoPPX, 71% in MitoPPN, 64% in ER-PPX, and 44% in CytoPPX cells, compared to the Wt samples. In MitoPPN cells, all the parameters assayed where decreased (except spare capacity), when compared to MitoPPX cells. Moreover, MitoPPX cells showed decreased respiration in comparison with the Wt cells, especially when we assayed maximal respiration (which was decreased by 36%) and spare capacity (which was decreased by 74%). The differences in the case of ER-PPX and CytoPPX cells, when compared with Wt samples, were of a lesser magnitude (Figure 7A). Contrary to the decrease in non-mitochondrial respiration and spare capacity which was observed in MitoPPX and MitoPPN cells, ER-PPX and CytoPPX cells showed an increase in these respiratory parameters, when compared to Wt. Specifically, non-mitochondrial respiration was increased by 44% in ER-PPX, and 69% in CytoPPX cells. Moreover, spare capacity was increased by 75% in ER-PPX and 62% in CytoPPX cells. All these percentages are calculated by normalizing the mean value of the specific raw values with the mean of the Wt samples.

### Expression of mitochondrial PPK deleteriously affects cell viability in SH-SY5Y cells

**FIGURE 8:**
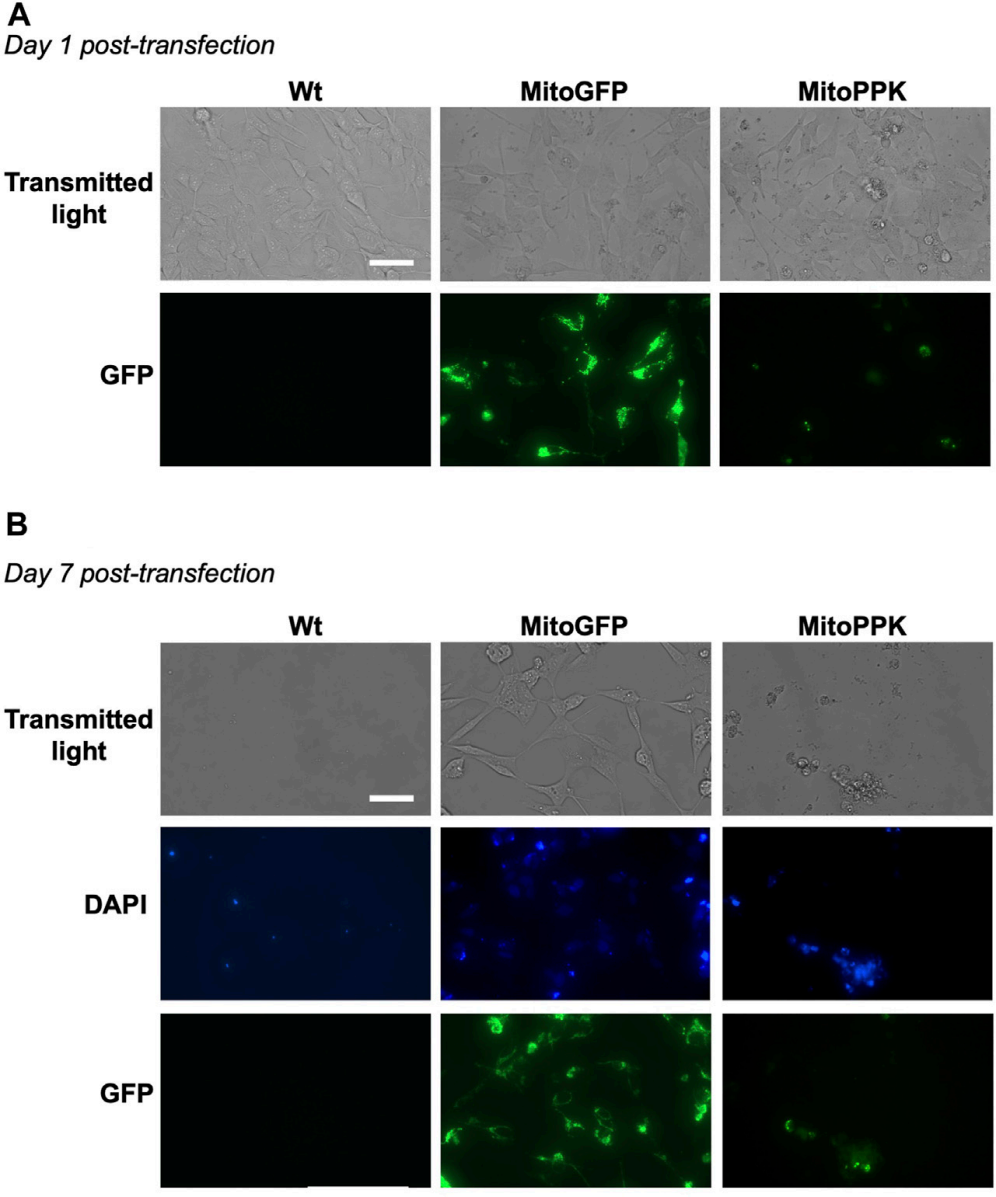
*Mitochondrial expression of PPK deleteriously affects the viability of SH-SY5Y cells.* Fluorescence images obtained using transmitted light and a GFP filter of MitoPPK cells. In this case, DAPI was visualized in the standard spectrum, as we used this dye to label the nuclei. **(A)**. on day one post-transfection, and **(B)**. on day seven post-transfection. MitoGFP cells were used as control. Note that the expression of MitoPPK deleteriously affects the viability of SH-SY5Y cells. Scale bar = 100 µm.

To increase the levels of mitochondrial polyP in our models, we used a mammalian vector to express in mitochondria of SH-SY5Y cells the PPK enzyme from *Saccharomyces cerevisiae* (MitoPPK cells). These cells expressed the plasmid immediately after transfection, as demonstrated by the presence of GFP (Figure 8A). Seven days post-transfection and under treatment with geneticin (selection antibiotic), cells were stained using DAPI (to label their nuclei, using the regular excitation and emission wavelengths) and imaged using a fluorescence microscope and the filters for DAPI and GFP. The images clearly demonstrated that the cells transfected with MitoPPK were not viable at this stage (Figure 8B). We repeated this assay two more times and obtained the same results. Therefore, we concluded that expression of MitoPPK is incompatible with cell viability in SH-SY5Y cells.

## Discussion

PolyP is an understudied polymer in mammalian cells. This is due, at least partially, to the dearth of models for its study. This lack of models is mostly a consequence of the poor understanding of the enzymes involved in its metabolism in mammalian cells. We have previously created stably transfected MitoPPX HEK293 and SH-SY5Y cells (Solesio et al., 2016b; Guitart-Mampel et al., 2022). However, MitoPPX cells do not allow to investigate the influence of chain length and diverse subcellular locations [both factors have been suggested to affect the role of mammalian polyP (Seidlmayer et al., 2019; Solesio et al., 2020)] on the effects of polyP. Therefore, just by using MitoPPX cells we are not able to obtain a comprehensive picture of the effects of polyP in mammalian physiology. Here, we present new cellular models which allow to study the effects of i) another enzyme involved in the metabolism of polyP (PPN), and ii) PPX expressed in other subcellular compartment, different from mitochondria.

The lack of well-defined protocols to study mammalian polyP is also an obstacle to advance the knowledge in this field. We and others have exploited the fluorescence shift induced by the DAPI-polyP complex (compared to the fluorescence spectra of the DAPI-nucleic acids complex [Kumble and Kornberg, 1995; Aschar-Sobbi et al., 2008)]. However, different protocols, have been proposed by multiple research groups to assay this fluorescence. This results in high variability of the data regarding the levels of mammalian polyP. In the methods section of this manuscript, we describe in detail a protocol that we have optimized for mammalian cells and that can be used to assay the effects of the enzymes involved in the metabolism of polyP in different subcellular compartments of these cells. The binding between DAPI and polyP could be non-specific under certain conditions (DAPI can also bind to inositol pyrophosphates and RNA, and not only to polyP (Aschar-Sobbi et al., 2008; Losito et al., 2009; Martin and Van Mooy, 2013; Kolozsvari et al., 2014)). However, our experiments using exogenous PPX, a very specific enzyme that degrades polyP (Akiyama et al., 1993; Wurst and Kornberg, 1994), show that our protocol specifically detects polyP.

Our data show that the targeted expression in mammalian cells of the enzymes involved in the degradation of polyP in microorganisms (PPX and PPN) is a valid strategy to introduce these proteins in different subcellular locations. Specifically, for PPX, for which antibodies are commercially available, our results show the high co-localization between this protein and the specific subcellular compartments that were targeted, assayed by Pearson’s coefficient and densitograms of representative areas. EM images showed fundamental differences in the structure of mitochondria from some of the cell lines that we have created, including CytoPPX, MitoPPX, and MitoPPN cells. In fact, while CytoPPX cells presented an elevated number of mitochondria containing electron dense areas, MitoPPX and MitoPPN cells showed high proportion of electron lucent mitochondria, compared to Wt cells. Some authors have shown that polyP accumulates in electron dense areas in diverse models (Ryan, 1969; LeFurgey et al., 1990; Ota et al., 2016). Conversely, the depletion of polyP could appear as electron lucent areas. It has also been described that alterations of mitochondrial inner membrane topology are associated with the increased presence of electron lucent mitochondria. These alterations cause downstream effects on key mitochondrial processes (Mannella, 2006). Therefore, depletion of mitochondrial polyP could induce inner mitochondrial membrane changes, which will affect bioenergetics, as observed in this study. Increased presence of electron dense mitochondria in CytoPPX cells could be a consequence of the depletion of cytoplasmic polyP in these cells. This could suggest an inter-organelle mechanism involved in the regulation of polyP, which needs to be investigated further.

Our cellular models are also a valid strategy to modify the endogenous levels of polyP in mammalian cells. In fact, our data also show that PPX and PPN are active and able to hydrolyze polyP, which aligns with the previous findings obtained with MitoPPX cells (Hambardikar et al., 2022). The only exception to this was found in ER-PPX cells, which do not show decreased levels of polyP in the ER, despite the presence of active PPX in this compartment. PolyP localization in the ER is not well described in literature, however the enrichment of polyP has been shown in ER from osteoblastic cell lines (SaOS-2) (Khong et al., 2020). In the same study, the authors showed depleted levels of polyP in the ER after transient transfections with the ER-PPX construct (Khong et al., 2020). The differences between these findings and ours could be a consequence of the use of cell types with substantially different energy metabolism (SaOS-2 vs. SH-SY5Y cells). Moreover, these different findings could also be explained by the short-term (transient transfection) vs. long-term (stable transfection) effects of PPX in polyP in the ER. In the mammalian cell, mitochondria and ER are in close contact, via the mitochondria-associated ER membranes (MAMs) (Raturi and Simmen, 2013). One of the main proteins involved in these junctions between mitochondria and ER is mitofusin 2 (Mfn2) (Filadi et al., 2015). Mfn2 is present on the outer mitochondrial membrane and the ER surface (de Brito and Scorrano, 2008). Specifically, it has been described that decreased Mfn2 increases the mitochondrial-ER tethering and cross-talk (Filadi et al., 2015). Parkin is another protein typically involved in these joint areas, whose regulation is linked to that of Mfn2 (Basso et al., 2018).

Our qPCR data show a sharp decrease in the expression of the genes that code for Mnf2 and Parkin proteins in ER-PPX cells. This could be associated with increased MAMs in response to polyP depletion in ER, probably as a compensatory mechanism to maintain the levels of polyP in ER. It is important to note that ER plays a crucial role in protein synthesis and folding, and that the role of polyP as a primordial chaperone has been previously described (Gray et al., 2014). Therefore, ER might need sustained levels of polyP to maintain proper protein homeostasis and cell viability. Moreover, mitochondria have been described by us and others as one of the preferred locations for polyP in mammalian cells (Abramov et al., 2007; Solesio et al., 2016b), and it has been proposed that polyP could be generated by the ATP synthase (Baev et al., 2020). This could further support our explanation of a compensatory effect involved in the maintenance of steady levels of polyP in the ER: polyP could be imported from mitochondria to ER, via MAMs, when the levels are decreased in ER by the expression of ER-PPX. This would produce no significant differences in the presence of polyP in ER from ER-PPX cells.

The qPCR data also show significant increased expression of SIRT3 in MitoPPX, MitoPPN, and ER-PPX cells, and even if not significant, a similar tendency is observed in CytoPPX cells. Furthermore, SOD2 expression is increased in SH-SY5Y cells with depleted mitochondrial polyP (MitoPPX and MitoPPN). SOD2 and Sirtuin 3 are mitochondrial proteins involved in the cellular stress response. Their activation is closely related to the status of mitochondrial physiology, especially to that of reactive oxygen species (ROS) generation (Flynn and Melov, 2013; Zhang et al., 2020). Therefore, the increased expression of SIRT3 and SOD2 could be interpreted as a cellular response to counteract the metabolic and oxidative stress induced by the depletion of polyP. In fact, increased cellular stress has already been described in MitoPPX cells (Solesio et al., 2021; Guitart-Mampel et al., 2022; Hambardikar et al., 2022). Interestingly, changes in much of the genes that we studied have been broadly demonstrated in the main neurodegenerative disorders, including Parkinson’s Disease and Amyotrophic Lateral Sclerosis (Tomkins et al., 2001; Dawson and Dawson, 2010; Belluzzi et al., 2012; Palomo et al., 2018; Vinciguerra et al., 2023).

Our attempts to create stably transfected SH-SY5Y MitoPPK cells were unsuccessful. While the transfection of SH-SY5Y cells with the MitoPPK plasmid was possible, and the plasmid was expressed in mitochondria (based on GFP fluorescence), MitoPPK cells became inviable soon after transfection. This opens interesting questions regarding the effects of the chain length of polyP in mammalian physiology. The current consensus is that the length of the polymer in these organisms is around a few tens of Pi, perhaps even less, and that this number is dependent on the specific cell type (Seidlmayer et al., 2019). For example, some authors have shown that, in human platelets, secreted polyP is around 60-100 Pi long (Ruiz et al., 2004). Other authors have shown that the length of polyP is significatively higher in mice, and that it can vary substantially between different mammalian cell lines. In fact, their data show that in these cells, polyP is found mostly in two clusters: between five and 15 Pi, and between 500 and 800 Pi. The relative amount of polyP in these clusters is very dependent on the specific cell line, with PC12, Jurkat, and NIH3T3 having the highest presence of short length polyP (Kumble and Kornberg, 1995). While the literature is scarce, the toxicity of long chain polyP in mammalian cells has already been suggested by some authors. For example, as previously mentioned, long chains of the polymer induce major effects in the transcriptome, proteome, and phosphatome of mammalian cells (Bondy-Chorney et al., 2020; Pirttiniemi et al., 2023), which could be associated with substantial changes in cell physiology, including viability. Furthermore, it has recently been published that increased release of polyP by astrocytes causes toxicity to motoneurons (Arredondo et al., 2022). Considering all this, our data suggest a low tolerance for longer chains of polyP in SH-SY5Y cells, which aligns with the limited bibliography available.

We further explored the effects of the length of polyP in mammalian cell physiology by using Seahorse technology. All cell lines show decreased OCR in response to treatment with FCCP. This demonstrate that these newly created models have appropriate mitochondrial membrane potential, which aligns with our previous findings in HEK293 MitoPPX cells (Solesio et al., 2021). Our Seahorse data also show that the transfection of SH-SY5Y cells with the MitoPPX construct has deleterious effects on mitochondrial bioenergetics, what corroborates our previous findings in HEK293 cells (Solesio et al., 2021). The observed drop is even more dramatic in the MitoPPN cells, which suggests that this effect is length-dependent. The differences between PPN and PPX in the cleavage of polyP, and the substrate-dependent specific enzyme activity of these two enzymes have been previously explored in other organisms (Andreeva et al., 2019). Specifically, the study shows that, in *S. cerevisiae*, PPX has similar enzymatic activity on long (polyP_208_) and short chain (polyP_3_) substrates. However, PPN has a higher enzymatic specificity by long chain polyP (polyP_208_), as compared to short chain (polyP_3_). All this has never been addressed in mammalian cells. The substrate specificity of the PPN enzyme could also explain the relatively higher DAPI-polyP signal observed in the enzymatic assay of the MitoPPN cells, as the degradation of polyP plateaus after a specific chain length of polyP is reached.

Considering all this, our data suggest that the effects of polyP in the maintenance of mitochondrial bioenergetics are highly chain length-dependent. This data aligns with our findings regarding loss of cell viability in MitoPPK cells. Moreover, the influence of the length of polyP in its effects on mammalian physiology has already been shown. For instance, in murine cardiac myocytes, short chain polyP activates the mPTP, while long chain polyP suppresses mPTP activation and enhances energy production and cell metabolism (Seidlmayer et al., 2019). Another study has reported that longer chains of polyP are more effective in the stimulation of amyloidogenic proteins to form fibrils, which has a protective effect on mammalian physiology (Gray et al., 2014; Cremers et al., 2016). In our models, mitochondrial respiration is not only affected by the depletion of mitochondrial polyP, but also by the modification of the length and the concentration of extra-mitochondrial levels of polyP (CytoPPX and ER-PPX cells). This, jointly with the data regarding the levels of polyP in the ER-PPX cells, suggests that polyP can bypass membranes, via an unknown mechanism. In fact, the ability of polyP to create channels in the membranes of bacteria has already been demonstrated (Moreno and Docampo, 2013); and the role of polyP as a structural component of the mammalian mitochondrial permeability pore has also been established (Seidlmayer et al., 2012a; Seidlmayer et al., 2012b).

Here, we present a toolkit of cellular models to study mammalian polyP. These models were created through stable expression of enzymes involved in polyP metabolism in different subcellular locations of SH-SY5Y cells. Our results show that the expression of these enzymes is, in fact, targeted to specific organelles. Moreover, these enzymes are active and their expression induces profound changes in both the morphology of mitochondria and the gene expression in the SH-SY5Y cells. We also demonstrate that SH-SY5Y have a low tolerance for long chains of polyP. Lastly, our data demonstrate the potent regulatory effects of mammalian mitochondrial polyP on bioenergetics, which appear to be affected by the specific length of polyP. This expands the regulatory role to polyP in other subcellular compartments. The use of our cellular models could expand the study of polyP, and contribute to a better understanding of this understudied, ancient polymer. Moreover, dysfunctional mitochondrial bioenergetics has been broadly demonstrated in many human pathologies (Galindo et al., 2012; Solesio et al., 2012; Solesio et al., 2013b; Patro et al., 2021). Therefore, a better understanding of the molecular mechanisms that regulate mitochondrial bioenergetics could contribute to increase our knowledge of the etiopathology of these diseases.

## Model sharing

Stably transfected cell lines will be shared under a biological Material Transfer Agreement (MTA). To ask for these cells, please email the corresponding author of this manuscript (m.solesio@rutgers.edu). The sharing of these biological samples will be contingent on the recipient agreeing to cite this manuscript.

## Data Availability

The raw data supporting the conclusion of this article will be made available by the authors, without undue reservation.
